# Strengthening integration of family planning with HIV/AIDS and other services: experience from three Kenyan cities

**DOI:** 10.1186/s12978-019-0715-8

**Published:** 2019-05-29

**Authors:** Raymond Mutisya, Jonesmus Wambua, Paul Nyachae, Mercy Kamau, Shalmali Radha Karnad, Mark Kabue

**Affiliations:** 1 Jhpiego Kenya, Nairobi, Kenya; 2 Jhpiego Baltimore, Baltimore, MD USA

**Keywords:** Family planning, HIV/AIDS, Service providers, Levels of integration, Unmet need

## Abstract

**Background:**

Kenya has made remarkable progress in integrating a range of reproductive health services with HIV/AIDS services over the past decade. This study describes a sub-set of outcomes from the Bill & Melinda Gates Foundation (BMGF)-funded Jhpiego-led Kenya Urban Reproductive Health Initiative (*Tupange*) Project (2010–2015), specifically addressing strengthening family planning (FP) integration with a range of primary care services including HIV testing and counselling, HIV care services, and maternal, newborn and child care.

**Methods:**

A cross-sectional study was conducted between August and October 2013 in the cities of Mombasa, Nairobi and Kisumu in Kenya to assess the level of FP integration across six other service delivery areas (antenatal care clinic, maternity wards, postnatal care clinic, child welfare clinic, HIV testing and counseling (HTC) clinics, HIV/AIDS services in comprehensive care clinics). The variables of interest were level of integration, provider knowledge, and provider skills. Routine program monitoring data on workload was utilized for sampling, with additional data collected and analyzed from twenty health facilities selected for this study, along with client exit interviews. Descriptive analysis and Chi-square/ Fishers Exact tests were done to explore relationships between variables of interest.

**Results:**

Integration of FP occurred in all the five service areas to varying degrees. Service provider FP knowledge in four service delivery areas (HTC clinic, antenatal clinic, postnatal clinic, and child welfare clinic) increased with increasing levels of integration. Forty-seven percent of the clients reported that time spent accessing FP services in the HTC clinic was reasonable. However, no FP knowledge was reported from service providers in HIV/AIDS comprehensive care clinics in all levels of integration despite observed provision of counseling and referral for FP services.

**Conclusions:**

Integration of FP services in other primary care service areas including HTC clinic can be enhanced through targeted interventions at the facility. A holistic approach to address service providers’ capacity and attitudes, ensuring FP commodity security, and creating a supportive environment to accommodate service integration is necessary and recommended. Additional studies are necessary to identify ways of enhancing FP integration, particularly with HIV/AIDS care services.

**Electronic supplementary material:**

The online version of this article (10.1186/s12978-019-0715-8) contains supplementary material, which is available to authorized users.

## Background

Unmet need for family planning (FP) continues to be a challenge, with 12% of women either married or in-union reporting unmet need in 2015 globally [1], 22% of them residing in the least developed countries [1]. Unmet need in Sub-Saharan Africa is highest, with double (24%) the global average [1]. This is compounded by a high HIV prevalence rate in the region [2]. 62–93% of pregnancies among the HIV infected women living in Sub-Saharan Africa are unintended [3, 4]. According to the 2014 Kenya Demographic and Health Survey (KDHS), 18% of women currently married or in union have unmet need for FP, with higher need in rural areas (20.2%) than in urban areas (13.4%) [5]. This represents marginal improvement from the 26% level of unmet need reported in 2009 [6]. The survey showed a decline in unwanted birth from 17 % to 10 % since the 2008–09 KDHS [6]. Kenya is among the 22 countries that collectively account for nearly 90% of all pregnant women living with HIV [7]. The consequences of unintended pregnancies can be profound putting women living with HIV at greater risk of death during the pregnancy and postpartum period than women without HIV [8]. Strengthening HIV and reproductive health (RH) service integration is one of the ten goals set forward in the 2013 UNAIDS report [9]. Studies in Kenya have shown that integrating FP and HIV services is acceptable, feasible and cost effective [10–15]. One potential benefit of integrated care is increased utilization of individual component health services [16]. Integration between RH and HIV services has been rolled out in Kenya as a strategy to create synergies in addressing missed opportunities in HIV prevention and care as well as in RH care across the service delivery levels [17]. Further efforts are however needed to promote uptake of FP in the service delivery system to accelerate progress towards addressing the unmet needs.

The desire to expand access and use of FP to all sexually active individuals at any time (and especially within the healthcare settings) provided the impetus for FP integration with other service delivery areas [18].

The key benefits of integrated models of service delivery are; improved quality of care and clinical outcomes, greater treatment engagement for patients who are either resistant to treatment or are difficult to reach in more conventional care models, and improved patient satisfaction and targeting of resources [19]. Integration is therefore key to meeting international and national development goals and targets, particularly the Sustainable Development Goal 3 [20]. This however is not without challenges which should be addressed to realize the full benefits of integration [21, 22].

The integration of health services has been defined in a variety of ways from both recipient or health system perspectives [23, 24]. Several models of FP integration with other service areas have been shown to work well [25]. One such model, the single visit approach, has been demonstrated to maximize resources through the use of common space, reducing staff costs, and lowering overheads [26].

In Kenya, deliberate efforts have been made to integrate RH services with HIV/AIDS and other services guided by a minimum package which lists the requirements for effective integration by level of care [27]. The goal of the package is to operationalize the *National Reproductive Health and HIV/AIDS Integration Strategy 2009* which laid down the framework for integrating RH and HIV services to provide more comprehensive, convenient, acceptable and cost effective RH and HIV/AIDS programs [17]. However, implementation experience based on the minimum package has not been documented.

The objective of this study was to assess integration of FP, HIV, and other primary health services (primarily maternal, newborn and child health) in high volume health facilities in three major cities in Kenya. The *Tupange* Project had led a range of FP interventions in facilities within these three locations, including specific interventions aimed at integration of FP into other service areas to decrease unmet need. The assessment was based on the definition of Kenya’s *National Minimum Package for RH and HIV Integrated Services* [27]. The three cities demonstrated a high level of unmet need for FP among women in 2009, ranging from 18% in the richest quintile in Nairobi to 41% in the poorest quintile in Mombasa [28].

## Methods

### Study design

A cross sectional design was utilized. The Johns Hopkins School of Public Health IRB (IRB No. 4993) and the Kenyatta National Hospital / University of Nairobi (KNH/UON) Ethics Review Committee approved the study.

### Study setting

The study was conducted between August and October 2013 in the three cities - Nairobi, Mombasa and Kisumu – as a component of the Bill & Melinda Gates Foundation (BMGF)-funded Jhpiego-led Kenya Urban Reproductive Health Initiative (*Tupange*) Project. The cities were selected on the basis that they accounted for over 50% of Kenya’s urban population of about 5 million, according to the 2009 Kenya Population and Housing Census [29]. Six service areas of interest were identified for assessment on service integration in the public and private health facilities. These areas are antenatal care (ANC) clinics, maternity wards, child welfare clinic (CWC), postnatal care (PNC) clinics, HIV/AIDS care services in comprehensive care clinics (CCCs), and HIV testing and counselling (HTC) clinics.

### Description of the Tupange project intervention

The *Tupange* project was a five-year project (2010–2015), implemented by a consortium of five partners: Jhpiego; Center for Communication Programs (CCP); Marie Stopes International (MSI); National Council for Population and Development (NCPD); and Pharm Access Africa Limited (PAAL) [30]. The project was initiated at a time when the national health efforts were focused on provision of HIV and primary health care services to the rural population, leaving the FP needs of the urban poor inadequately addressed, despite the rapid urbanization of the major cities in Kenya [30]. *Tupange*’s goal was to increase contraceptive prevalence rate by 20 percentage points among the urban poor in five urban cities in Kenya [31].

The project implemented multiple interventions to strengthen health systems and improve access to quality FP services at the facility and community level by equipping facilities, and training and mentoring service providers. The *Tupange* project supported public and private health facilities through scheduled visits to facilities by a team of experts to enhance the uptake of long and permanent contraceptive methods, ensure FP commodity security, and advocate for increased resource allocation towards reproductive health services [31].

**Fig. 1:**
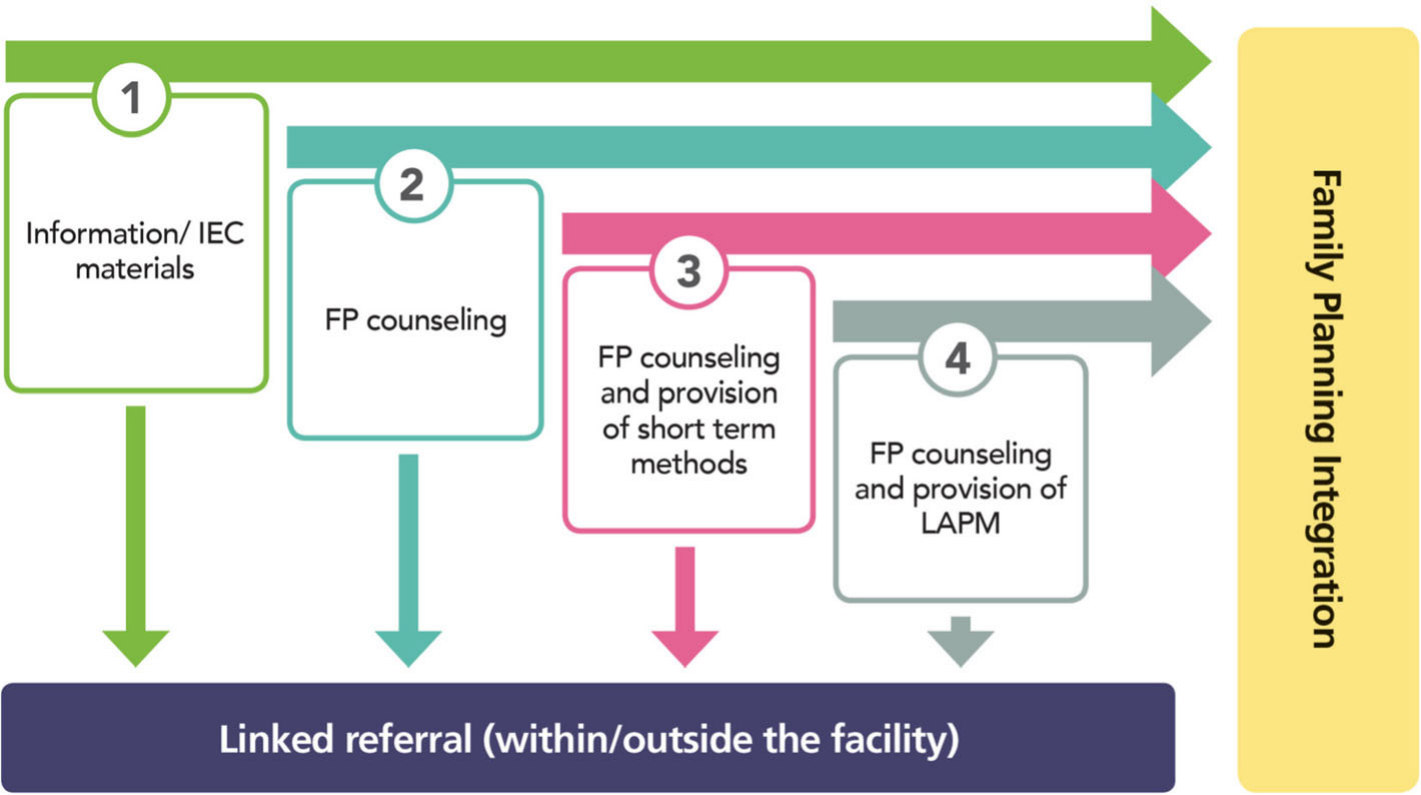
Provider Initiated FP (PIFP) model

The *Tupange* project developed a Provider Initiated FP (PIFP) model (Fig. 1) where service providers actively initiated integrated discussions on FP and HIV/AIDS with the clients, counselled appropriately, and offered both FP method administration and HIV testing in an integrated manner. Tools to document FP integration in other service areas were incorporated in the routine reporting system and referral notes were used to refer clients within or outside the facility. The PIFP model is based on a continuum of FP service delivery across four levels (Fig. 1): Client referrals within and outside the facility was done to ensure that all clients received multiple services (as needed) in any single visit.

### Sampling and study population

Twenty of the 69 *Tupange*-supported high volume health facilities in three of the five *Tupange* cities were selected using probability proportionate to size sampling: nine in Nairobi, six in Mombasa, and five in Kisumu. The other two cities – Machakos and Kakamega – were scale-up sites thus not included in the first 2 years of the project. Criteria for selection was based on city, volume/workload, management category (hospital, clinic, health center), and managing authority/ownership (public/private/municipal). All of the facilities had a daily workload in all the service areas of approximately 50–100 clients based on data gathered for the 6 months preceding the survey.

#### Service providers

At participating health facilities, between five to six providers were selected for interview, one from each service areas of interest. All service providers in the 20 health facilities working in the six service areas were eligible to participate. Whenever there was more than one eligible service provider, the in-charge was selected and approached to participate.

#### Clients

Two clients aged 15–54 years seeking services at any of the six service areas in 20 health facilities were selected through systematic sampling at the end of the visit for client exit interview at the respective service areas. Every fifth client was selected as this sampling technique provided research assistants (RAs) adequate time to complete the interview and embark on another one while at the same time limiting selection bias.

### Data collection

Integration study data was collected by trained RAs between August and October 2013. The interview tools for each service area comprised of mixed (open and closed ended) questions on demographics, FP knowledge, experiences providing FP services, barriers to FP provision, and perceptions on how long it took clients to access services at the various service delivery points.

Every client participating in the study was asked about the FP information and counselling they received from service providers during the visit, as well as their perspective of waiting time and integration of services. Overall, the data collection period for both service providers and clients lasted for 2 months. Interviews were conducted in locations within the facility where audio and visual privacy was guaranteed.

Written consent to participate was obtained from all study participants.

### Data analysis

The study investigators defined levels of service integration as follows: Category 0: No integration; Category 1: Provision of FP information, education and communication (IEC) materials and counselling only and referral; and Category 2: FP counselling and provision of short-term and long-term methods. Short term methods are a range of contraceptive methods that are user dependent and need to be taken on a daily, weekly or monthly basis, and include all FP methods other than the long acting and reversible contraceptives (such as Intrauterine devices and contraceptive implants) and permanent methods. Descriptive statistics were used to summarize categorical data through counts and frequencies. Comparison of the following components in the three levels of integration was done: level of service FP knowledge, training and skills, and barriers to FP service provision. Chi-squared and Fisher’s Exact Tests were used to elucidate differences between, with a *p*-value significance level of < 0.05.

## Results

### Demographics of service providers

There were only eight services providers who served in maternity while the others had an average of 19 respondents, and therefore not included in the analysis. One hundred and three service providers were interviewed in the five service areas excluding maternity area. A majority (94.2%) were nurses, female (92.2%), and the median (interquartile range [IQR]) age was 33 (27–44) years. The median (IQR) duration after graduation was 7 (5–16) years, with most providers working in their health facility for a median (IQR) duration of 32 (17–38) months. Ninety-six client exit interviews were completed. The demographic data were not collected from participants since, the interviews were anonymous. Table 1 summarizes the demographic characteristics of the service providers.

**Table 1:** Description of the study population – Service providers

Characteristic	Median (IQR)	Number	Percent
Age (years): *N* = 103	33 (27–44)		
Duration since graduation (years): N = 103	7 (5–16)		
Work experience (Months): N = 103	32 (17–38)		
Sex (Female)		95	92.2%
Cadre			
General practitioner/ Doctor		2	1.9%
Clinical Officer		4	3.9%
Nurse		97	94.2%
Total		103	100%

### Integration of services by service area

There was evidence of FP integration – particularly counseling and provision of a method (Category 2) – with all HTC, ANC, PNC, and CWC. At ANC, only FP counselling (Category 1) was done, with provision of FP methods expected to take place after delivery, including issuing of condoms. Only FP counselling and referral was done in the HIV/AIDS care services/ CCC since at the time of the survey, FP services were not provided in CCCs.

The level of FP knowledge among service providers varied greatly between the different service areas. There was an apparent increase in the level of FP knowledge between non-integrated health facilities (Category 0) to health facilities with higher levels of integration (Category 2), although the differences were not statistically significant. No FP knowledge was reported from service providers in HIV/AIDS care services/ CCC in all levels of integration (Table 2).

**Table 2:** Family planning knowledge and skills of service providers by level of integration

Variable Description	N	Category 0:No Integration	Category 1 Integration	Category 2 Integration	p-value
A: Service providers with adequate knowledge on FP					
*HTC*	18	*n* = 8 (0%)	*n* = 4 (25%)	*n* = 6 (33%)	0.275^a^
HIV/AIDS care services	19	n = 1 (0%)	n = 4 (0%)	*n* = 14 (0%)	Not done
*ANC*	19	n = 4 (50%)	*n* = 13 (69%)	*n* = 2 (100%)	0.599^a^
*PNC*	20	*n* = 3 (33%)	n = 1 (100%)	*n* = 16 (75%)	0.455^a^
*CWC*	19	n = 8 (75%)	n = 8 (75%)	n = 3 (100%)	1.000^a^
B: Service provider training and skills					
Skills on providing short term FP methods	103	*n* = 26 (96%)	*n* = 34 (97%)	*n* = 43 (93%)	0.850^b^
Skills on providing LARC FP methods	103	n = 26 (85%)	n = 34 (85%)	n = 43 (81%)	0.944^b^
Ever received training in FP	103	n = 26 (92%)	n = 34 (88%)	n = 43 (100%)	0.048^b^
Received training in FP during the 12 months preceding the survey	103	n = 26 (81%)	n = 34 (68%)	n = 43 (72%)	0.522^b^
Has access to guidelines for providing FP services	89	*n* = 19 (95)	*n* = 30 (83)	*n* = 40 (85)	0.612^b^
Has good knowledge of FP guidelines for providing FP services	83	*n* = 17 (71%)	*n* = 28 (65%)	*n* = 36 (66%)	0.901^b^
Discusses with clients about need for family planning	95	*N* = 23 (87%)	*N* = 31 (100%)	*N* = 41 (80%)	0.020
C: Barriers to providing FP services					
Experienced FP supplies/ commodities shortages	103	n = 26 (62%)	n = 34 (44%)	n = 43 (30%)	0.0382^b^
Inappropriate facility - confidentiality	103	n = 26 (12%)	n = 34 (3%)	n = 43 (5%)	0.431^b^
Heavy workload	103	n = 26 (19%)	n = 34 (24%)	n = 43 (26%)	0.915^b^

^a^Fisher's Exact test p-value; ^b^Chi-square test p-value

Service providers had skills on provision of short term in almost the same proportion across the three categories of FP integration i.e. from 96% (category 0), 97% (category 1) and 93% (category 2) for short-term methods. With regard to long acting and reversible contraceptive methods (LARCs), the skills level was lower than for short-term methods; 85% (category 0), 85% (category 1), and to 81% (category 2). These differences were not statistically significant. However, all service providers in category 2 facilities had ever received training in FP compared to 88% (category 1), and 92% (category 0), *p* = 0.048. Another notable finding was that although service providers reported discussing FP matters with clients routinely, the extent to which this happened varied with the degree of integration; 87% (Category 0), 100% (category 1), and 80% (category 3), *p* = 0.020.

Lack of FP supplies / commodities was reported as a barrier to provision of FP services by service providers in all health facilities but at a varying degree with a lower proportion of those in the more integrated health facilities reporting it as a barrier; 62% (category 0), 44% (category 1) and 30% (category 2), *p* = 0.038. The proportions of service providers in facilities across the various integration levels reporting inappropriate space in facilities / lack of confidentiality or heavy workload as barriers were generally low (3–26%). These differences were not statistically significant (Table 2).

### Client perspective of waiting time and integration of services

Two hundred and thirty-eight client exit interviews were conducted; 90.1% were women, 78.8% were married/cohabiting, median (IQR) age was 27 (24–32) years, and 48.3% of them had either no formal education or primary level (8 years). Majority of the clients (85.3%) reported that they received all the services they needed during the visit (Table 3).

**Table 3:** Client perceptions on time spent at different service areas

Perception of duration	HTC (*N* = 15)	ANC (*N* = 20)	PNC (N = 16)	CWC (N = 31)	HIV/AIDS Care services/CCC(N = 14)	Average (*N* = 96)
Reasonable	46.7%	45.0%	56.2%	54.8%	50.0%	50.5%
Short	20.0%	10.0%	12.5%	0.0%	7.1%	9.9%
Long	33.3%	40.0%	31.3%	45.2%	42.9%	38.6%
Missing	0%	5.0%	0%	0%	0%	1.0%
Total	100%	100%	100%	100%	100%	100%

Overall, 50.5% of the clients across the five service delivery points indicated that the time spent by was reasonable, while 38.6% considered the duration long, and only 9.9% responded that the duration was short (Table 3). Clients reported spending about 1 h to access all the services they needed during their visit, although a few reported spending up to as much as 6 h. Results from the observation of 20 clients showed that on average most clients visited 4–5 delivery points in a single visit and spent on average a median (IQR) time of 80 (55–138) minutes during the visit.

Nearly all clients (97.1%) reported that one service provider was not able to meet all their needs during the visit thus they were referred either to another section within the health facility or outside the health facility. Twenty percent of the clients at HTC service area reported that they had spent a short time compared to clients in other service areas who reported longer waiting time (Table 3).

## Discussion

The WHO and PEPFAR definitions of integration of health services provide a comprehensive picture of integration from the recipient and health system perspectives. For this to happen, health facility infrastructure must be receptive, with adequate resources to enable provision of multiple, appropriate, cost effective, and timely services needed by the clients. Health facility assessments had been done at the beginning of the *Tupange* project and were not part of this survey. The findings of our study provide insights into the various service provider and client satisfaction factors that are relevant to integration of FP services with HIV and other primary care services, and thus contributes to the body of knowledge in this field, which is not very well understood. The findings suggest that integration of FP services with other critical health service areas such as HTC or HIV/AIDS care services in CCCs can occur with concerted efforts in training and mentoring of providers to improve knowledge of integrated service offerings, strengthening supply chain support, and improving health service infrastructure.

The study highlighted several service provider factors that influence the level of FP integration, with service provider FP knowledge increasing with the category of FP integration across in all service delivery areas. However, no FP knowledge was reported from service providers in HIV/AIDS care service in CCCs in all levels of integration. This was unexpected since provision of condoms for dual protection was done as part of the HIV/AIDS package of care in all study sites [27]. Actions and attitudes of service providers towards provision of services are important since they determine the care they offer to clients and shape the outcome of that care. Additional studies are necessary to understand this observation and identify ways of addressing FP integration challenges in the HIV/AIDs care service area.

The proportion of service providers with skills in provision of short-term methods and LARC remained the same across the three categories of integration, suggesting that factors other than ability to offer short-term methods and LARC played an important role in limiting provision of these services across HTC, ANC, PNC, and CWC. Minimal access and knowledge on FP guidelines by service providers was observed in integration Category 2 facilities when compared to facilities with no integration further strengthening the likelihood of other factors influencing FP integration. This highlights important question on what is required to motivate service providers who are knowledgeable with the necessary skills and reference materials to promote integration. Understanding these issues is critical in creating an enabling environment for integration.

It is apparent that empowering service providers with knowledge and skills in a variety of relevant service delivery areas is a prerequisite for a successful integration of FP with other service areas. However, while training is necessary for quality service delivery and performance improvement, it is not sufficient to bring about integration.

According to WHO, the performance of health workers depends not only on their competence (knowledge, skills) but also on their availability (retention and presence), their motivation and job satisfaction, as well as the availability of infrastructure, equipment and support systems, such as the management, information systems, resources and accountability systems that are in place [32].

Service providers’ attitudes towards FP integration is equally important for a successful integration. Studies in Kenya indicate a lack of pre-service and in-service training of health personnel on service integration contribute to negative attitudes exhibited by some service providers, especially nurses [33]. Provider ownership of the integration process and understanding what needs to be integrated and how to integrate is also important. This highlights the need for regular follow up initiatives to provide support supervision post training and mentorship sessions. This is also important for examining not only the effectiveness of the project in changing knowledge, attitudes and behaviours of service providers, but also the durability of its impact [34].

Service providers in facilities with no integration reported lack of FP supplies/ commodities and inappropriate facility infrastructure for privacy and confidentiality as the main barriers to provision of FP services. In contrast, service providers in facilities at category 2 of integration reported heavy workload as the main barrier to provision of FP services. Other studies have shown that providers may be hesitant to provide integrated services due to their own biases and lack of information [21].

The findings of this study are suggestive that additional time is required to adequately counsel each client to enable them make an informed family planning decision. This in turn increases staff workload as well as client waiting time especially where staffing levels are low thus making some providers avoid integrating FP when they are offering other services, choosing rather to address patients’ explicit or immediate needs only, before attending to the next client in order to reduce the waiting time. While understandable given time and workload constraints, this approach creates missed opportunities to counsel clients on FP. Similar findings are reported by Okundi et al. in their 2009 study which found that, where staff felt too burdened with sick and complicated patients to spend the time to discuss FP, and also found that integrating services can result in additional time needed to serve each client [35].

The benefits of integration however outweigh any challenges if the full benefits of addressing missed opportunities to offer timely interventional services are factored in. Our study showed, that on average, 50% of the clients reported spending reasonable time in the service areas they had sought services despite varying degrees of integration in the facilities. This may be an indication that the benefits of accessing extra services as a result of FP integration in a given service area in addition to the primary reason for seeking medical services was worthy spending more time with the service provider.

The findings on our study contribute towards enriching available literature on FP integration. It highlights important issues that should be considered for any successful FP integration and clearly demonstrates that training of service providers alone cannot guarantee FP integration. Hence, programs should pursue holistic approaches that addresses capacity shortfalls and attitude of the service providers as well as strengthening the human resource systems that create a supportive working environment in addition to equipping facilities to accommodate the extra services.

This study has a limitation of cross-sectional design as data were gathered at only one time-period thus only presents a snapshot of the situation. Additionally, data were based on self-report and not complemented with direct observation of practice. However, the use of face-to-face interviews to collect data in the real world situations provides valuable insights into what transpired during the consultation visits. The findings could have been enriched by incorporating direct observations in the study which future studies can incorporate in the design in order to document the actual practice of integration of FP and HIV services.

## Conclusions

The benefits of FP use extend beyond the individual to the population at large and to the children. However, as missed opportunities for contraception persist, with corresponding increase in unplanned and unwanted pregnancies, higher maternal morbidity and mortality rates are inevitable, moving countries further away from achieving the Sustainable Development Goal 3. Strengthening integration of FP services in other service areas is a strategy when properly implemented targets to reduce these missed opportunities. A holistic approach to address service providers’ capacity and attitudes, ensuring FP commodity security and creating a supportive environment to accommodate service integration is recommended. Additional research is needed to assess documentation of FP integration in the context of competing demands on service providers.

A French translation of this article has been included as Additional file 1.

A Portuguese translation of the abstract has been included as Additional file 2.

## Additional file

Additional file 1: Translation of this article into French. (PDF 357 kb)

Additional file 2: Translation of the abstract of this article into Portuguese. (PDF 105 kb)

## Abbreviations

ANC: Antenatal care
CCC: Comprehensive Care Clinics
CWC: Child welfare clinic
FP: Family planning
HIV/AIDS: Human immunodeficiency virus / Acquired immune deficiency syndrome
HTC: HIV testing and counselling
IEC: Information education communication
IQR: Interquartile range
IRB: Institutional review board
KDHS: Kenya demographic health survey
LAMP: Long acting and permanent method
M&E: Monitoring and evaluation
MCH: Maternal child health
PEPFAR: The President’s Emergency Plan For AIDS Relief
PIFP: Provider initiated family planning
PNC: Postnatal care
RH: Reproductive health
WHO: World Health Organization

## References

[1] Darroch Jacqueline E. (2013). Trends in contraceptive use. Contraception.

[2] UNFPA (2016). UNFPA annual report 2016: Millions of lives transformed.

[3] Homsy Jaco, Bunnell Rebecca, Moore David, King Rachel, Malamba Samuel, Nakityo Rose, Glidden David, Tappero Jordan, Mermin Jonathan (2009). Reproductive Intentions and Outcomes among Women on Antiretroviral Therapy in Rural Uganda: A Prospective Cohort Study. PLoS ONE.

[4] Schwartz SR, Rees H, Mehta S, Venter WDF, Taha TE, Black V (2012). High incidence of unplanned pregnancy after antiretroviral therapy initiation: findings from a prospective cohort study in South Africa. PLoS One.

[5] Kenya National Bureau of Statistics IM (2014). Demographic and Health Survey 2014.

[6] Ojiambo Robert, Gibson Alexander R., Konstabel Kenn, Lieberman Daniel E., Speakman John R., Reilly John J., Pitsiladis Yannis P. (2013). Free-living physical activity and energy expenditure of rural children and adolescents in the Nandi region of Kenya. Annals of Human Biology.

[7] UNAIDS (2012). Together we will end AIDS.

[8] Calvert C, Ronsmans C (2013). The contribution of HIV to pregnancy-related mortality: a systematic review and meta-analysis. Aids.

[9] UNAIDS (2013). GLOBAL REPORT: UNAIDS report on the global AIDS epidemic 2013.

[10] Newmann Sara J., Mishra Kavita, Onono Maricianah, Bukusi Elizabeth A., Cohen Craig R., Gage Olivia, Odeny Rose, Schwartz Katie D., Grossman Daniel (2013). Providers’ Perspectives on Provision of Family Planning to HIV-Positive Individuals in HIV Care in Nyanza Province, Kenya. AIDS Research and Treatment.

[11] Shade SB, Kevany S, Onono M, Ochieng G, Steinfeld RL, Grossman D (2013). Cost, cost-efficiency and cost-effectiveness of integrated family planning and HIV services. Aids.

[12] Cohen CR, Grossman D, Onono M, Blat C, Newmann SJ, Burger RL (2017). Integration of family planning services into HIV care clinics: results one year after a cluster randomized controlled trial in Kenya. PLoS One.

[13] Steinfeld Rachel L., Newmann Sara J., Onono Maricianah, Cohen Craig R., Bukusi Elizabeth A., Grossman Daniel (2013). Overcoming Barriers to Family Planning through Integration: Perspectives of HIV-Positive Men in Nyanza Province, Kenya. AIDS Research and Treatment.

[14] Newmann SJ, Grossman D, Blat C, Onono M, Steinfeld R, Bukusi EA (2013). Does integrating family planning into HIV care and treatment impact intention to use contraception? Patient perspectives from HIV-infected individuals in Nyanza Province, Kenya. Int J Gynecol Obstet.

[15] Haberlen SA, Narasimhan M, Beres LK, Kennedy CE (2017). Integration of family planning services into HIV care and treatment services: a systematic review. Stud Fam Plan.

[16] Church K, Warren CE, Birdthistle I, Ploubidis GB, Tomlin K, Zhou W (2017). Impact of integrated services on HIV testing: a nonrandomized trial among Kenyan family planning clients. Stud Fam Plan.

[17] Kenya R of (2009). National Reproductive Health and HIV and AIDS Integration Strategy.

[18] Foundation MG. London Summit on Family Planning. 2012;2012:1–19 Accessed 5 Feb 2018.

[19] Atun R, de Jongh TE, Secci FV, Ohiri K, Adeyi O, Car J (2011). Integration of priority population, health and nutrition interventions into health systems: systematic review. BMC Public Health.

[20] Good health and well-being: why it matters? 2017. Accessed 10 Feb 2018

[21] Maharaj P, Cleland J (2005). Integration of sexual and reproductive health services in KwaZulu-Natal, South Africa. Health Policy Plan.

[22] Winestone Lena E., Bukusi Elizabeth A., Cohen Craig R., Kwaro Daniel, Schmidt Nicole C., Turan Janet M. (2012). Acceptability and feasibility of integration of HIV care services into antenatal clinics in rural Kenya: A qualitative provider interview study. Global Public Health.

[23] Brief TT, Goals MD (2008). Making health systems work.

[24] January F. PEPFAR guidance on integrating prevention of mother to child transmission of HIV , Maternal , Neonatal , and Child Health and Pediatric HIV Services 2011. Accessed 29 Jan 2018

[25] IATT (2014). Compendium of Case Studies HIV and Sexual and Reproductive Health Programming: Innovative Approaches to Integrated Service Delivery.

[26] Gribble J, Foreman MIA. BUREAU MATERNAL AND CHILD HEALTH CARE : 2011;1999. https://assets.prb.org/pdf11/familyplanning-maternal-child-health.pdf.

[27] Health KM of. Minimum Package for Reproductive Health (RH) and HIV Integrated Services 2012. Accessed 5 Feb 2018.

[28] Kenya urban reproductive health initiative (Tupange); Report of the 2010 baseline household survey 2011. Accessed 29 Jan 2018.

[29] Statistics KNB of (2010). 2009 Kenya Population and Housing Census.

[30] Keyonzo N, Nyachae P, Kagwe P, Kilonzo M, Mumba F, Owino K (2015). From project to program: Tupange’s experience with scaling up family planning interventions in urban Kenya. Reprod Health Matters.

[31] Muthamia M, Owino K, Nyachae P, Kilonzo M, Kamau M, Otai J (2016). The Tupange project in Kenya: a multifaceted approach to increasing use of long-acting reversible contraceptives. Glob Heal Sci Pract.

[32] USAID, UNICEF W. Towards universal access: scaling up HIV services for women and children in the health sector. Progress Report. 2008;2008 Accessed 10 Feb 2018.

[33] Dieleman M, Harnmeijer JW. Improving health worker performance : in search of promising practices. Hum Resour Health. 2006:77. https://www.who.int/hrh/resources/improving_hw_performance.pdf.

[34] Mockiene V, Suominen T, Välimäki M, Razbadauskas A (2010). Impact of intervention programs on nurses’ knowledge, attitudes, and willingness to take care of patients with human immunodeficiency virus/acquired immunodeficiency syndrome: a descriptive review. Med.

[35] Okundi B, Aloo-Obunga C, Sanders R, Shepherd C, Green C (2009). Rapid assessment on policy and operational barriers to the integration of FP/RH/HIV services in Kenya | UNESCO HIV and Health education clearinghouse.

