# Supplementary material for: Strengthening integration of family planning with HIV/AIDS and other services: experience from three Kenyan cities
Source: Reprod Health. 2019 May 29;16(Suppl 1):62. doi: 10.1186/s12978-019-0715-8 (PMC6538540; doi:10.1186/s12978-019-0715-8)
Supplement: Supplementary file 1 — Translation of this article into French. (PDF 357 kb) [file 12978_2019_715_MOESM1_ESM.pdf]

Reproductive Health 2019, 16 (Suppl 1) [translated from the original publication]

Renforcement de l'intégration de la planification familiale dans les services de prise en charge du VIH/SIDA et d'autres services : l'expérience de trois villes kenyanes

Raymond Mutisya[1*], Jonesmus Wambua[1], Paul Nyachae[1], Mercy Kamau[1], Shalmali Radha Karnad[1], Mark Kabue[2],

[1]Jhpiego Kenya, Nairobi, Kenya
[2]Jhpiego Baltimore, Baltimore, MD, USA

* Coordonnées de l'auteur correspondant :Raymond Mutisya, Raymond.Mutisya@jhpiego.org; aymondmutisya@yahoo.com

Adresse électronique des auteurs :
Jonesmus Wambua, Jonesmus.Wambua@jhpiego.org
Paul Nyachae, Paul.Nyachae@jhpiego.org
Mercy Kamau, Mercy.Kamau@jhpiego.org
Shalmali Radha Karnad, Radha.Karnad@jhpiego.org
Mark Kabue, Mark.Kabue@jhpiego.org

Résumé :

Contexte

Au cours des dix dernières années, le Kenya a réalisé des progrès considérables dans l'intégration de certains services de santé reproductive dans les services de prise en charge du VIH/SIDA. Cette étude décrit un sous-ensemble de résultats du projet Kenya Urban Reproductive Health Initiative (*Tupange*) (2010-2015) financé par la fondation Bill & Melinda Gates (BMGF) et mené par Jhpiego, étudiant principalement le renforcement de l'intégration de la planification familiale (PF) dans un certain nombre de services de soins primaires dont le dépistage du VIH et le conseil, les services de prise en charge du VIH, et de soins maternels, néonatals et infantiles.

Méthodes

Une étude transversale menée d'août à octobre 2013 dans les villes de Mombasa, Nairobi et Kisumu au Kenya a évalué le niveau d'intégration de la PF dans six autres domaines de prestation de services (centres de soins prénatals, maternités, centres de soins post-natals, centres de protection de l'enfance, centres de dépistage du VIH et de conseil (DVC), services de prise en charge du VIH/SIDA dans des

centres de soins complets). Les variables d'intérêt étaient le niveau d'intégration, le niveau de connaissances du prestataire de soins et la qualification du prestataire de soins. Les données des programmes de surveillance de routine sur la base du volume de travail ont été utilisées pour l'échantillonnage, ainsi que des données supplémentaires collectées auprès de vingt établissements de santé sélectionnés pour cette étude, et analysées, avec les entretiens de sortie des patients. Une analyse descriptive et des tests du Chi-carré/ exacts de Fisher ont été réalisés pour étudier la relation entre les variables d'intérêt.

Résultats

L'intégration de la PF avait lieu à différents degrés dans chacun des cinq domaines de services. Le niveau de connaissances en matière de PF du prestataire de soins dans quatre domaines de service (centre de DVC, centre de soins prénatals, centre de soins post-natals, centre de protection de l'enfance) augmentait à mesure que le niveau d'intégration augmentait. Quarante-sept pour cent des patients indiquait que le temps passé à accéder aux services de PF dans la clinique de DVC était raisonnable. Cependant, aucune connaissance de la PF n'a été indiquée pour les prestataires de soins dans les centres de soins complets prenant en charge le VIH/le SIDA quel que soit le degré d'intégration, même la prestation de conseils et le renvoi à des services de PF ont été observés.

Conclusions

L'intégration de services de PF dans d'autres domaines de services de soins primaires, dont les centres de DVC peut être renforcée par des interventions ciblées dans l'établissement. Une approche holistique axée sur la compétence et les attitudes des prestataires de soins, assurant la sécurité de fourniture de la PF, et créant un environnement favorable à la réception de l'intégration des services est nécessaire et recommandée. Des études supplémentaires sont nécessaires pour identifier les manières de renforcer l'intégration de la PF, notamment avec les services de prise en charge du VIH/du SIDA.

Mots-clés : Planification familiale, VIH/SIDA, prestataires de soins, niveaux d'intégration, besoin non satisfait.

Contexte

Le besoin non satisfait de planification familiale (PF) continue à représenter un défi, 12 % des femmes mariées ou en couple dans le monde indiquant un besoin non satisfait en 2015[1], 22 % d'entre elles résidant dans les pays les moins développés[1]. Le besoin non satisfait est le plus élevé en Afrique subsaharienne, qui enregistre le double (24 %) de la moyenne mondiale[1]. Ceci est aggravé par un taux élevé de prévalence du VIH dans la région[2]. 62 à 93 % des grossesses parmi les femmes infectées par le VIH vivant en Afrique subsaharienne ne sont pas programmées [3, 4]. Selon l'étude Kenya Demographic and Health Survey (KDHS) de 2014, 18 % des femmes actuellement mariées ou en couple ont un besoin non satisfait de PF, le besoin étant plus marqué en zone rurale (20,2 %) qu'en zone urbaine (13,4 %)[5]. Ceci représente une amélioration marginale par rapport au besoin non satisfait de 26 % observé en 2009[6]. L'étude a montré une baisse des naissances non programmées de 17 % à 10 % depuis l'étude KDHS de 2008-2009[6]. Le Kenya fait partie des 22 pays totalisant environ 90 % de toutes les femmes enceintes vivant avec le VIH[7]. Les conséquences des grossesses non programmées peuvent être graves, et mettre davantage en danger la vies des femmes vivant avec le VIH pendant la grossesse et la période post-partum que celle des femmes sans VIH[8]. Le renforcement de l'intégration des services de prise en charge du VIH et de santé reproductive (SR) est l'un des dix objectifs énoncés dans le rapport ONUSIDA de 2013[9]. Les études menées au Kenya ont montré que l'intégration de la PF dans les services VIH est acceptable, réalisable et économique[10–15]. L'un des avantages potentiels de l'intégration des services est l'augmentation de l'utilisation des différents composants des services de santé[16]. L'intégration entre les services de SR et VIH a été déployée au Kenya en tant que stratégie visant à créer des synergies pour remédier aux occasions manquées dans la prévention et la prise en charge du VIH et dans la SR à tous les niveaux de service[17]. Des efforts supplémentaires sont

cependant nécessaires pour promouvoir l'implantation de la PF dans le système de prestation de services pour accélérer les avancées en matière de réponse aux besoins non satisfaits.

Le souhait d'étendre l'accès à la PF et son utilisation à toutes les personnes sexuellement actives à tout moment (et en particulier dans l'environnement médical) ont donné une impulsion à l'intégration de la PF dans d'autres domaines de services[18].

Les avantages principaux des modèles intégrés de services sont : une amélioration de la qualité des soins et des résultats cliniques, un engagement accru dans le traitement des patients qui sont soit résistants au traitement soit difficiles à atteindre dans des modèles de soins plus conventionnels, et une amélioration de la satisfaction du patient et du ciblage des ressources[19]. Par conséquent, l'intégration est essentielle pour atteindre les objectifs internationaux et nationaux de développement, et en particulier l'Objectif de développement durable 3[20]. Ceci comporte cependant des défis qui doivent être relevés afin d'atteindre pleinement les objectifs de l'intégration[21, 22].

L'intégration des services de santé a reçu de nombreuses définitions, tant du point de vue du destinataire que du système de santé[23, 24]. Plusieurs modèles d'intégration de la PF à d'autres domaines de services ont montré leur efficacité[25]. L'un de ces modèles, l'approche « visite unique » a montré qu'elle permettait de maximiser les ressources par l'utilisation d'un espace commun, réduisant les frais de personnel et les frais généraux [26].

Au Kenya, des efforts ont été faits pour intégrer les services de SR et les services VIH/SIDA et autres guidés par un ensemble minimum contenant les exigences d'intégration efficace par niveau de soins[27]. L'objectif de l'ensemble est de rendre opérationnelle la *Stratégie nationale d'intégration de la santé reproductive et du VIH/SIDA 2009*, qui définissait le cadre de l'intégration des services de SR et de VIH pour fournir des programmes de SR et VIH/SIDA plus complets, pratiques, acceptables et

économiques[17]. Cependant, l'expérience de mise en œuvre à partir de l'ensemble minimum n'a pas été documentée.

L'objectif de cette étude était d'évaluer l'intégration des services de PF, VIH/SIDA et d'autres soins de santé primaires (soins maternels, néonatals et infantiles primaires) dans les établissements de santé à volume important dans trois grandes villes du Kenya. Le projet *Tupange* a mené à une série d'interventions de PF dans les établissements de ces zones, y compris à des interventions spécifiques visant à intégrer la PF dans d'autres domaines de services pour réduire le besoin non satisfait. L'évaluation reposait sur la définition du *National Minimum Package for RH and HIV Integrated Services (Ensemble minimum national de services intégrés pour la SR et la prise en charge du VIH)* du Kenya[27]. En 2009, les trois villes présentaient un niveau élevé de besoin non satisfait en PF auprès des femmes, allant de 18 % dans le quintile le plus riche à Nairobi à 41 % dans le quintile le plus pauvre à Mombasa[28].

Méthodes

Conception de l'étude

Une étude transversale a été réalisée. L'IRB de la Johns Hopkins School of Public Health (IRB n°4993) et le Ethics Review Committee du Kenyatta National Hospital / University of Nairobi (KNH/UON) ont approuvé l'étude.

Configuration de l'étude

L'étude a été menée d'août à octobre 2013 dans les trois villes (Nairobi, Mombasa et Kisumu), et faisait partie du projet Kenya Urban Reproductive Health Initiative (*Tupange*) financé par la fondation Bill & Melinda Gates (BMGF) et mené par Jhpiego. Les villes ont été sélectionnées sur la base du fait qu'elles représentaient plus de 50 % de la population urbaine du Kenya comptant environ 5 millions de

personnes, selon le recensement de la population et de l'habitat du Kenya de 2009[29]. Six domaines de services ont été identifiés pour l'évaluation de l'intégration des services dans les établissements de santé publics et privés. Il s'agit des centres de soins prénatals (SPN), des maternités, des centres de protection de l'enfance (CPE), des centres de soins postnatals (SPoN), des services de prise en charge du VIH/SIDA dans les centres de soins complets (CSC) et des centres de dépistage du VIH et de conseil (DVC).

Description de l'intervention du projet Tupange

Le projet *Tupange* était un projet d'une durée de 5 ans (2010-2015) mis en place par un groupe de cinq partenaires : Jhpiego ; Center for Communication Programs (CCP) ; Marie Stopes International (MSI) ; National Council for Population and Development (NCPD) ; et Pharm Access Africa Limited (PAAL)[30]. Le projet a débuté alors que les efforts de santé du gouvernement se concentraient sur la fournitures de soins de prise en charge du VIH et de santé primaire à la population rurale, laissant les besoins en PF de la population urbaine et pauvre non satisfaits, malgré l'urbanisation rapide des grandes villes du Kenya[30]. L'objectif de *Tupange* était d'augmenter le taux de prévalence de la contraception de 20 points de pourcentage auprès des pauvres des zones urbaines dans cinq villes du Kenya[31].

Le projet a déployé plusieurs interventions pour renforcer les systèmes de santé et améliorer l'accès à des services de PF de qualité aux niveaux des établissements et communautaire en équipant les équipements et en formant et en encadrant les prestataires de soins. Le projet *Tupange* soutenait les établissements de santé publics et privés à l'aide de visites programmées des établissements par des équipes d'experts pour améliorer le recours à des méthodes de contraception permanente et à longue durée d'action, assurer la sécurité des lieux de PF et promouvoir l'augmentation de l'allocation des ressources aux services de santé reproductive[31].

Le projet *Tupange* a mis au point un modèle de PF à l'initiative du prestataire de soins (PFIP) (Figure 1) dans lequel les prestataires de soins lançaient activement des discussions intégrées sur la PF et le VIH/SIDA avec les patients, conseillaient de manière appropriée et proposaient de manière intégrée une méthode d'administration de la méthode de PF et un dépistage du VIH. Des outils permettant de documenter l'intégration de la PF dans d'autres domaines de services ont été incorporés dans les systèmes d'information habituels et des notes de référence ont été utilisées pour référer les patients l'établissement ou en dehors de celui-ci. Le modèle PFIP repose sur un continuum des services de PF sur les quatre niveaux (Figure 1) : Les patients ont été référés dans l'établissement et en dehors de ce dernier pour s'assurer qu'ils bénéficiaient tous de plusieurs services (selon leurs besoins) lors d'une visite unique.

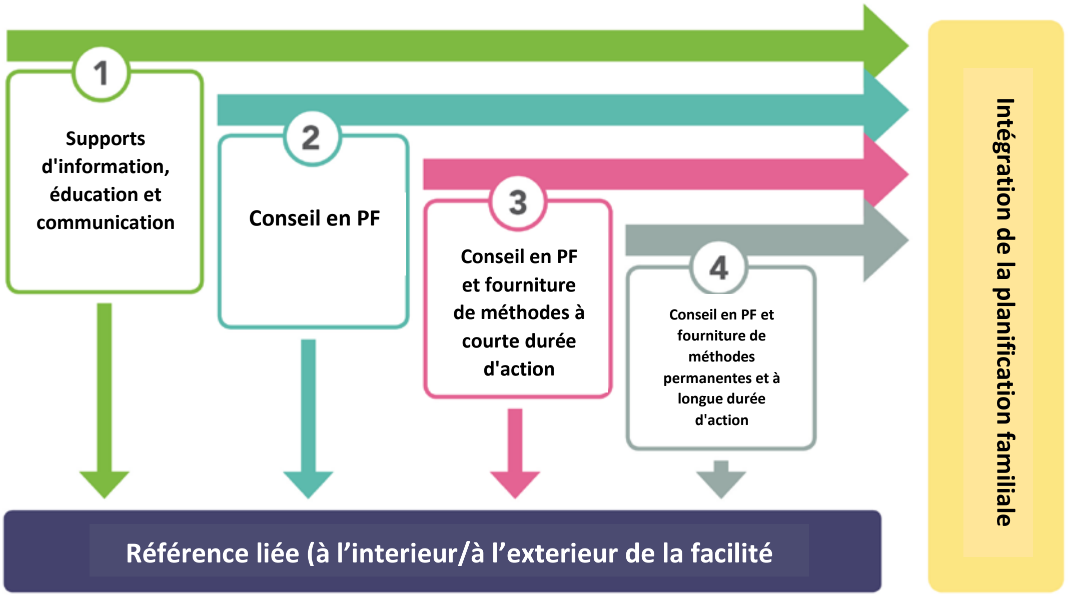

Figure 1 : Modèle de PF à l'initiative du prestataire de soins (PFIP)

Échantillonnage et population de l'étude

20 des 69 établissements de santé à volume important dans trois des cinq villes faisant partie du projet *Tupange,* ont été sélectionnés par échantillonnage avec probabilité proportionnelle à la taille : neuf à Nairobi, six à Mombasa et cinq à Kisumu. Les deux autres villes, Machakos et Kakamega, étaient des

sites de mise à l'échelle et n'ont pas été incluses les deux premières années du projet. Le critère de sélection reposait sur la ville, le volume/la charge de travail, la catégorie de gestion (hôpital, clinique, centre de santé) et l'autorité de gestion/le propriétaire (public/privé/municipal). Les données collectées sur les 6 mois précédant l'étude indiquent que tous les sites avaient un volume de travail quotidien de 50 à 100 patients dans tous les domaines de services.

Prestataires de soins : dans les établissements de santé participants, cinq à six prestataires de soins étaient sélectionnés pour un entretien, un dans chaque domaine de services d'intérêt. Tous les prestataires de soins des 20 établissements de santé travaillant dans les six domaines de services étaient éligibles. En cas de présence de plus d'un prestataire de soins éligible, le prestataire responsable était sélectionné et invité à participer.

Patients : deux patients de 15 à 54 ans à la recherche de services dans l'un des six domaines de services dans les 20 établissements de santé ont été sélectionnés par échantillonnage systématique à la fin de leur visite pour un entretien de sortie du patient dans les domaines de service correspondants. Un patient sur cinq a été sélectionné car cette technique d'échantillonnage laissait aux assistants de recherche (AR) suffisamment de temps pour terminer l'entretien et en commencer un autre tout en limitant le biais de sélection.

Collecte des données

Les données de l'étude d'intégration ont été collectées par des AR formés, d'août à octobre 2013. Les outils d'entretien pour chaque domaine de service comprenaient des questions mixtes (ouvertes et fermées) sur l'aspect démographique, la connaissance en matière de PF, l'expérience dans la fourniture de services de PF, les barrières à la prestation de PF, et les perceptions sur le temps nécessaire aux patients pour accéder aux services dans les différents lieux de prestation.

Chaque patient participant à l'étude a répondu à des questions concernant les informations sur la PF et le conseil qu'ils avaient reçu des prestataires de soins pendant leur visite, ainsi que de leur perception des temps d'attente et de l'intégration des services. Dans l'ensemble, la période de collecte des données pour les prestataires de soins et les patients a duré deux mois. Les entretiens ont été menés dans des locaux des établissements assurant le respect de la vie privée au niveau audio et visuel.

Tous les participants à l'étude ont donné leur consentement écrit à participer.

Analyse des données

Les enquêteurs ont défini les niveaux d'intégration des services de la manière suivante : Catégorie 0 : Aucune intégration ; Catégorie 1 : Fourniture d'informations sur la PF, supports d'éducation et de communication (IEC) ainsi que conseil uniquement et orientation ; et catégorie 2 : Conseil en PF et fourniture de méthodes à courte durée d'action et à longue durée d'action. Les méthodes à courte durée d'action sont des méthodes de contraception dépendant de l'utilisateur et devant être prises sur une base quotidienne, hebdomadaire ou mensuelle, et comprennent toutes les méthodes de PF autres que les moyens de contraception réversible et à longue durée d'action (dispositifs intra-utérins et implants contraceptifs) et que les méthodes permanentes. Des statistiques descriptives ont été utilisées pour synthétiser les données par catégorie au moyen de totaux et de fréquences. Les éléments suivants ont été comparés dans les trois niveaux d'intégration : niveau de connaissance du service de PF, formation et compétences, et barrières à la fourniture de services de PF. Des tests Chi-carré et exacts de Fischer ont été utilisés pour expliquer les différences entre eux, avec un niveau de signification de la valeur p <°0,05.

Résultats

Données démographiques des prestataires de soins

Seuls 8 prestataires de soins opéraient en maternité, tandis que les autres services avaient une moyenne de 19 répondants, et n'ont par conséquent pas été inclus dans l'analyse. 103 prestataires de soins ont été interviewés dans les cinq domaines de services, à l'exclusion du domaine Maternité. Il s'agissait en majorité de personnels infirmiers (94,2 %), de sexe féminin (92,2 %) et l'âge médian (écart interquartile [EI]) était de 33 (27 – 44) ans. La durée médiane (EI) était de 7 (5-16) après obtention du diplôme, la plupart des prestataires de soins travaillant dans leur établissement de santé pour une durée médiane (EI) de 32 (17-38) mois. 96 entretiens de sortie des patients ont été réalisés. Les données démographiques des participants n'ont pas été collectées car les entretiens étaient anonymes. Le Tableau 1 résume les caractéristiques démographiques des prestataires de soins.

Tableau 1 : Description de la population de l'étude – Prestataires de soins

Intégration des services par domaine de services

Il existait des preuves d'intégration de la PF, en particulier du conseil et de l'indication d'une méthode (catégorie 2) avec tous les DVC, SPN, SPoN et CPE. Dans les centres de SPN, seul le conseil en PF (catégorie 1) était offert, l'indication des méthodes de PF étant supposée avoir lieu après la naissance, avec distribution de préservatifs. Dans les centres de prise en charge du VIH/SIDA dans les CSC, seuls un conseil en PF et l'orientation étaient fournis, car à l'époque de l'étude, les services de PF n'étaient pas fournis dans les CSC.

Le niveau de connaissance de la PF auprès des prestataires de soins variait considérablement entre les différents domaines de services. Une augmentation apparente du niveau de connaissances en matière de PF a été notée entre les établissements de santé non intégrés (catégorie 0) et les établissements de santé présentant un niveau plus élevé d'intégration (catégorie 2), bien que ces différences ne soient pas significatives d'un point de vue statistique. Aucune connaissance de la PF n'a été indiquée par les

prestataires de soins dans les centres de prise en charge du VIH/SIDA dans les CSC à tous les niveaux d'intégration (Tableau 2).

Tableau 2 : Connaissances et compétences des prestataires de soins en matière de planification familiale selon le niveau d'intégration

Les prestataires de soins possédaient des compétences dans l'indication d'une méthode à court terme dans une proportion quasiment identique dans les trois catégories d'intégration de la PF, à savoir de 96 % (catégorie 0), 97 % (catégorie 1) et 93 % (catégorie 2). Pour ce qui concerne les moyens de contraception à long terme réversible (LARC), le niveau de compétences était plus faible que pour les méthodes à court terme, à savoir 85 % (catégorie 0), 85 % (catégorie 1) et 81 % (catégorie 2). Ces différences n'étaient pas significatives d'un point de vue statistique. Cependant, tous les prestataires de soins des établissements de catégorie 2 avaient reçu une formation à la PF, contre 88 % (catégorie 1) et 92 % (catégorie 0), p=0,048. L'un des autres résultats significatifs était que, bien que les prestataires de soins indiquent qu'ils abordent habituellement le sujet de la PF avec les patients, la portée de cette discussion variait avec le niveau d'intégration : 87 % (catégorie 0), 100 % (catégorie 1) et 80 % (catégorie 3), p=0,020.

Le manque de fournitures/produits de PF a été indiqué comme une barrière à la fourniture de services de PF par les prestataires de soins dans tous les établissements de santé, mais à un niveau variable, une plus faible proportion des prestataires de soins travaillant dans les établissements de santé plus intégrés l'indiquant comme une barrière : 62 % (catégorie 0), 44 % (catégorie 1) et 30 % (catégorie 2), p=0,038. Le nombre de prestataires de soins dans les établissements à tous les niveaux d'intégration indiquant des locaux inadaptés / un manque de confidentialité ou une charge de travail importante comme des barrières était généralement faible (3 % - 26 %). Ces différences n'étaient pas significatives d'un point de vue statistique (Tableau 2).

Perception des temps d'attente et de l'intégration des services par les patients

Deux-cent trente-huit entretiens de sortie de patients ont été menés, 90,1 % étaient des femmes, 78,8 % étaient mariés/en couple, l'âge médian (EI) était de 27 (24-32) ans, et 48,3 % d'entre-eux n'avaient pas été scolarisés ou avaient été scolarisés au premier degré (8 ans). La majorité des patients (85,3 %) indiquaient avoir reçu tous les services dont ils avaient besoin pendant la visite (Tableau 3).

Tableau 3 : Perception par les patients du temps passé dans les différents domaines de services

Dans l'ensemble, 50,5 % des patients dans les cinq lieux de fourniture des services indiquaient que le temps passé était raisonnable, tandis que 38,6 % le trouvaient long, et seuls 9,9 % répondaient qu'il était court (Tableau 3). Les patients ont indiqué avoir passé environ une heure pour accéder à tous les services dont ils avaient besoin pendant leur visite, même si certains ont indiqué avoir passé jusqu'à 6 heures. Il ressort de l'observation de 20 patients que la plupart des patients se rendaient en moyenne dans 4 à 5 lieux de service au cours d'une même visite et passaient en moyenne une durée médiane (EI) de 80 (55-138 minutes) pendant la visite.

Presque tous les patients (97,1 %) indiquaient qu'un prestataire de soins n'était pas en mesure de répondre à tous leurs besoins pendant la visite et qu'ils étaient donc orientés vers un autre service dans l'établissement de santé ou en dehors de ce dernier. Vingt pour cent des patients du domaine de service DVC indiquaient qu'ils avaient passé peu de temps par rapport aux patients d'autres domaines de services ayant fait état de temps d'attente plus longs (Tableau 3).

Discussion

Les définitions de l'OMS et du PEPFAR de l'intégration des services de santé fournit un cadre complet de l'intégration, du point de vue du destinataire et du système de santé. Pour ce faire, l'infrastructure de l'établissement de santé doit être réceptive, avec des ressources appropriées pour permettre la

prestation des services nécessaires aux patients : multiples, adaptés, économiques et en temps voulu. Les évaluations des établissements de santé ont été réalisées au début du projet *Tupange* et ne faisaient pas partie de la présente étude. Les résultats de notre étude donnent un aperçu des différents facteurs de satisfaction des prestataires de soins et des patients pertinents pour l'intégration des services de PF dans les services de prise en charge du VIH et d'autres services de soins primaires, et contribuent ainsi à accroître les connaissances dans ce domaine, qui n'est pas encore très bien compris. Les résultats laissent à penser que l'intégration des services de PF à d'autres domaines de services de santé critiques tels que les services de DVC ou de prise en charge du VIH/SIDA dans les CSC peut être réalisée au moyen d'efforts concertés de formation et d'encadrement des prestataires de soins pour améliorer les connaissances en matière d'offre de services intégrés, en renforçant le soutien de la chaîne d'approvisionnement et en améliorant l'infrastructure des services de santé.

L'étude a mis en évidence plusieurs facteurs côté prestataires de soins qui influencent le niveau d'intégration de la PF, les connaissances du prestataire de soins en matière de PF augmentant avec la catégorie d'intégration de la PF dans tous les domaines de services. Cependant, aucune connaissance de la PF n'a été indiquée par les prestataires de soins dans les centres de prise en charge du VIH/SIDA dans les CSC à tous les niveaux d'intégration. Ceci était surprenant étant donné que des préservatifs sont fournis pour la protection des deux partenaires dans le cadre du programme de prise en charge du VIH/SIDA dans tous les établissements ayant participé à l'étude[27]. Les actions et les attitudes des prestataires de soins face à la fourniture de services sont importantes car elles déterminent les soins qu'ils offrent à leurs patients et influencent les résultats de ces soins. Des études supplémentaires sont nécessaires pour comprendre cette observation et identifier des manières de répondre aux défis de l'intégration de la PF, notamment dans le domaine de service de la prise en charge du VIH/du SIDA.

La proportion de prestataires de soins ayant des compétences dans l'indication de méthodes à courte durée d'action et de LARC était la même dans les trois catégories d'intégration, ce qui laisse à penser

que des facteurs autres que l'aptitude à offrir des méthodes à courte durée d'action et des LARC jouaient un rôle important dans la limitation de la fourniture de ces services dans les DVC, les CPN, les SPoN et les CPE. Un accès et des connaissances minimaux des directives de PF de la part des prestataires de soins ont été observés dans les sites de catégorie 2 d'intégration par rapport aux sites sans intégration, ce qui renforce la probabilité de l'influence d'autres facteurs sur l'intégration de la PF. Ceci met en évidence une question importante sur les facteurs de motivation des prestataires de soins informés disposant des compétences nécessaires et du matériel de référence pour promouvoir l'intégration. Il est crucial de comprendre ces problèmes pour créer un environnement favorable à l'intégration.

Il est manifeste que fournir aux prestataires de soins des connaissances et des compétences dans les différents domaines de services concernés est une condition nécessaire à la réussite de l'intégration de la PF dans d'autres domaines de services. Cependant, alors que la formation est nécessaire pour la fourniture de services de qualité et l'amélioration des performances, elle ne suffit pas à susciter l'intégration.

Selon l'OMS, la performance des professionnels de la santé ne dépend pas uniquement de leurs compétences (connaissances, aptitudes) mais aussi de leur disponibilité (rétention et présence), de leur motivation et de leur satisfaction au travail, ainsi que de la disponibilité de l'infrastructure, des équipements et des systèmes de soutien tels que les systèmes de gestion et informatiques, les ressources et les systèmes de comptabilité en place[32].

Les attitudes des prestataires de soins face à l'intégration de la PF sont également importantes pour une intégration réussie. Les études menées au Kenya indiquent que le manque de formation avant le service et pendant le service concernant l'intégration des services favorise les attitudes négatives montrées par certains prestataires de soins, notamment les infirmières[33]. La maîtrise du processus d'intégration par

le prestataire de soins et la compréhension des éléments à intégrer et de la manière de les intégrer sont également importantes. Ceci met en évidence le besoin d'initiatives de suivi régulier pour fournir une assistance et un accompagnement post-formation et des sessions d'encadrement. Ceci est également important pour étudier l'efficacité du projet dans l'évolution des connaissances, des attitudes et des comportements des prestataires de soins, mais aussi la durabilité de son impact[34].

Les prestataires de soins dans les établissements sans intégration ont indiqué le manque de fournitures/produits de PF et l'infrastructure inadaptée de l'établissement pour le respect de la vie privée et la confidentialité comme les barrières principales à la fourniture de services de PF. En revanche, les prestataires de soins dans les établissements ayant une intégration de catégorie 2 ont indiqué la charge de travail importante comme la barrière principale à la fourniture de services de PF. D'autres études ont montré que les prestataires de soins peuvent hésiter à fournir des services intégrés en raison de leurs propres préjugés et de leur manque d'information[21].

Les résultats de cette étude suggèrent qu'il faut plus de temps pour conseiller correctement chaque patient et lui permettre de prendre une décision de planification familiale éclairée. Ceci augmente la charge de travail du personnel et le temps d'attente des patients, notamment en cas de faibles effectifs, ce qui pousse certains prestataires de soins à ne pas intégrer la PF quand ils offrent d'autres services, et à choisir de répondre uniquement aux besoins exprimés ou immédiats des patients, avant de passer au patient suivant pour réduire le temps d'attente. Cette approche, bien que compréhensible étant donné les contraintes de temps et de charge de travail, est source d'occasions manquées de conseil aux patients sur leur PF. L'étude menée par Okundi et al. en 2009 a obtenu des résultats similaires, et a montré que, quand le personnel se sent trop surchargé par des cas complexes de patients malades, il ne prend pas le temps d'aborder la PF, et que l'intégration des services peut permettre de dégager le temps supplémentaire nécessaire pour servir chaque patient[35].

Cependant, les avantages de l'intégration sont plus importants que les défis si les avantages de remédier aux occasions manquées d'offrir des services d'intervention à temps sont pleinement pris en compte. Notre étude a démontré qu'une moyenne d'environ 50 % des patients passaient un temps raisonnable dans les domaines de services souhaités malgré différents niveaux d'intégration dans les établissements. Ceci peut indiquer que les avantages de pouvoir accéder à des services supplémentaires comme résultat de l'intégration de la PF dans un domaine de services donné, en plus de la raison primaire de l'accès au service médical en question, justifiaient le fait de passer plus de temps avec le prestataire de soins.

Les résultats de notre étude contribuent à enrichir la littérature actuelle sur l'intégration de la PF. Ils mettent en évidence les problèmes importants à prendre en compte pour une intégration réussie de la PF et démontrent clairement que la formation des prestataires de soins ne peut pas garantir à elle seule l'intégration de la PF. Par conséquent, les programmes doivent adopter une approche holistique qui réponde aux pénuries de capacité et à l'attitude des prestataires de soins et qui renforce les systèmes de ressources humaines créant un environnement de travail positif, et qui équipe également les établissement pour qu'ils puissent accueillir ces services supplémentaires.

Cette étude est limitée par sa conception transversale, car les données ont été collectées sur une seule période et ne fournissent ainsi qu'un instantané de la situation. Par ailleurs, les données reposaient sur des auto-évaluations et n'étaient pas complétées par une observation directe de la pratique. Cependant, le recours aux entretiens individuels pour collecter les données en situation réelle fournit un aperçu précieux de ce qui se passait lors des visites de consultation. Ces résultats auraient pu être complétés en intégrant à l'étude des observations directes, action que pourraient inclure des études futures afin de documenter la pratique actuelle de l'intégration de la PF et des services de prise en charge du VIH.

Conclusions

Les avantages de la PF ne se limitent pas aux individus, mais s'étendent à la population dans son ensemble et aux enfants. Cependant, tant que les occasions manquées de contraception persistent, avec une augmentation correspondante de grossesses non planifiées et non souhaitées, une morbidité et un taux de mortalité maternelle plus élevés sont inévitables, et continuent à éloigner les pays de l'Objectif 3 de Développement durable. Le renforcement de l'intégration des services de PF dans d'autres domaines de services est une stratégie pour réduire ces occasions manquées si elle est correctement mise en œuvre. Une approche holistique axée sur la compétence et les attitudes des prestataires de soins, assurant la sécurité de fourniture de la PF, et créant un environnement favorable à la réception de l'intégration des services est recommandée. Des recherches supplémentaires sont nécessaires pour évaluer la documentation de l'intégration de la PF dans le contexte d'exigences concurrentes posées envers les prestataires de soins.

Liste des abréviations

SPN : Soins prénatals **CPE :** Centre de protection de l'enfance **CSC :** Centre de soins complets **PF :** Planification familiale **VIH/SIDA :** Virus de l'immunodéficience humaine / Syndrome d'immunodéficience acquise **DVC** : dépistage du VIH et conseil **IEC :** Information, communication et éducation **EI :** Écart interquartile **IRB :** Institutional review board (comité d'éthique de la recherche) **KDHS :** Kenya demographic health survey (enquête démographique et de santé au Kenya) **LAPM :** Long acting and permanent method (méthode permanente et à longue durée d'action) **SMI :** Santé maternelle et infantile **S&E :** Suivi et évaluation **PEPFAR :** Plan d'urgence présidentiel américain d'aide d'urgence à la lutte contre le SIDA **PFIP :** PF à l'initiative du prestataire de soins **SPoN :** Soins postnatals **SR :** Santé reproductive **OMS :** Organisation mondiale de la Santé

Concernant ce supplément

Cet article a été publié comme partie de l'ouvrage *Reproductive Health*, Volume 16 Supplement 1, 2019: Effective Integration of Sexual Reproductive Health and HIV Prevention, Treatment, and Care Services across sub-Saharan Africa: Where is the evidence for program implementation?
Le supplément a été publié dans le cadre d'une collaboration entre *Reproductive Health* et *BMC Public Health.* L'intégralité du contenu, avec les versions en français, en portugais et en anglais, est disponible en ligne :
https://bmcpublichealth.biomedcentral.com/articles/supplements/volume-19-supplement-1
et
https://reproductive-health-journal.biomedcentral.com/articles/supplements/volume-16-supplement-1

Déclarations

Approbation éthique et accord de participation

L'étude a été approuvée par l'IRB de la Johns Hopkins School of Public Health (IRB n°4993) et l'Ethics Review Committee du Kenyatta National Hospital / University of Nairobi (KNH/UON).

Accord de publication

Non applicable

Disponibilité des données et matériels

Les données créées et/ou analysées pendant cette étude ne sont pas disponibles au public pour des raisons de confidentialité mais sont disponibles auprès de l'auteur correspondant sur demande raisonnable.

Conflits d'intérêts

Tous les auteurs déclarent ne pas avoir de conflits d'intérêts

Financement

Cette intervention et cette étude ont été possibles grâce au généreux soutien financier de la Fondation Bill & Melinda Gates (BMGF). Le contenu de cette étude est sous la responsabilité de Jhpiego Kenya et ne reflète pas nécessairement l'opinion de la BMGF. La publication de cet article a été financée par la BMGF.

Le supplément de la revue est rendu possible grâce au soutien généreux du peuple américain via la United States Agency for International Development (USAID) en partenariat avec le Fonds des Nations unies pour la population (FNUAP) et le Programme commun des Nations Unies sur le VIH/SIDA (ONUSIDA).

Les opinions exprimées dans la présente publication sont celles des auteurs et ne reflètent pas nécessairement les politiques officielles de l'USAID, du FNUAP ou de l'ONUSIDA, la mention des dénominations de ministères ou d'organismes n'implique pas non plus l'aval du gouvernement américain, du FNUAP ou de l'ONUSIDA.

Contributions des auteurs

Paul Nyachae et Mark Kabue se sont occupés de la conception et de l'élaboration de cette étude ; Mark Kabue et Jonesmus Wambua ont analysé les données ; Raymond Mutisya, Paul Nyachae, Mark Kabue, Mercy Kamau et Shalmali Radha Karnad ont contribué à la rédaction du manuscrit. Tous les auteurs ont participé à la révision critique du manuscrit et en ont approuvé la version finale.

Remerciements

Nous remercions particulièrement le ministère de la Santé du Kenya pour nous avoir permis d'accéder aux données des établissements, le personnel de Jhpiego qui a participé à la collecte des données, Kenneth Owino pour son assistance lors de l'analyse des données, Eunice Omanga, Elaine Charurat et Lindsay Breithaupt pour leur révision et leurs suggestions sur la version initiale.

Références

[1] United Nation, Department of Economic and Social Affairs PD. Trends in contraceptive use Worldwide 2015. 2015. doi:10.1016/j.contraception.2012.08.029. Accessed January 29, 2018

[2] UNFPA. UNFPA annual report 2016: Millions of lives transformed 2016:6–20. Accessed February 5, 2018

[3] Homsy J, Bunnell R, Moore D, King R, Malamba S, Nakityo R, et al. Reproductive intentions and outcomes among women on antiretroviral therapy in rural Uganda: A prospective cohort study. PLoS One 2009;4. doi:10.1371/journal.pone.0004149.

[4] Schwartz SR, Rees H, Mehta S, Venter WDF, Taha TE, Black V. High incidence of unplanned pregnancy after antiretroviral therapy initiation: Findings from a prospective cohort study in south africa. PLoS One 2012;7:1–8. doi:10.1371/journal.pone.0036039.

[5] Kenya National Bureau of Statistics IM. Demographic and Health Survey 2014. 2014. Accessed January 29, 2018

[6] Kenya National Bureau of Statistics (KNBS); ORC Macro. Kenya Demographic and Health Survey 2008-09. Heal (San Fr 2010:1–314. doi:10.3109/03014460.2013.775344. Accessed January 29, 2018

[7] UNAIDS. Together we will end AIDS. 2012. Accessed February 5, 2018

[8] Calvert C, Ronsmans C. The contribution of HIV to pregnancy-related mortality: A systematic review and meta-analysis. Aids 2013;27:1631–9. doi:10.1097/QAD.0b013e32835fd940.

[9] UNAIDS. GLOBAL REPORT: UNAIDS report on the global AIDS epidemic 2013. 2013. doi:JC2502/1/E. Accessed February 5, 2018

[10] Newmann SJ, Mishra K, Onono M, Bukusi EA, Cohen CR, Gage O, et al. Providers' perspectives on provision of family planning to HIV-positive individuals in HIV care in Nyanza province, Kenya. AIDS Res Treat 2013;2013. doi:10.1155/2013/915923.

[11] Shade SB, Kevany S, Onono M, Ochieng G, Steinfeld RL, Grossman D, et al. Cost, cost-efficiency and cost-effectiveness of integrated family planning and HIV services. Aids 2013;27:87–92. doi:10.1097/QAD.0000000000000038.

[12] Cohen CR, Grossman D, Onono M, Blat C, Newmann SJ, Burger RL, et al. Integration of family planning services into HIV care clinics: Results one year after a cluster randomized controlled trial in Kenya. PLoS One 2017;12:1–15. doi:10.1371/journal.pone.0172992.

[13] Steinfeld RL, Newmann SJ, Onono M, Cohen CR, Bukusi EA, Grossman D. Overcoming barriers to family planning through integration: Perspectives of HIV-positive men in Nyanza province, Kenya.

AIDS Res Treat 2013;2013. doi:10.1155/2013/861983.

[14] Newmann SJ, Grossman D, Blat C, Onono M, Steinfeld R, Bukusi EA, et al. Does integrating family planning into HIV care and treatment impact intention to use contraception? Patient perspectives from HIV-infected individuals in Nyanza Province, Kenya. Int J Gynecol Obstet 2013;123:e16–23. doi:10.1016/j.ijgo.2013.08.001.

[15] Haberlen SA, Narasimhan M, Beres LK, Kennedy CE. Integration of Family Planning Services into HIV Care and Treatment Services: A Systematic Review. Stud Fam Plann 2017;48:153–77. doi:10.1111/sifp.12018.

[16] Church K, Warren CE, Birdthistle I, Ploubidis GB, Tomlin K, Zhou W, et al. Impact of Integrated Services on HIV Testing: A Nonrandomized Trial among Kenyan Family Planning Clients. Stud Fam Plann 2017;48:201–18. doi:10.1111/sifp.12022.

[17] Kenya R of. National Reproductive Health and HIV and AIDS Integration Strategy 2009:1–52. Accessed January 29, 2018

[18] Foundation MG. London Summit on Family Planning , July 2012 2012:1–19. Accessed February 5, 2018

[19] Atun R, de Jongh TE, Secci F V, Ohiri K, Adeyi O, Car J. Integration of priority population, health and nutrition interventions into health systems: systematic review. BMC Public Health 2011;11:780. doi:10.1186/1471-2458-11-780.

[20] Good health and well-being: why it matters? 2017. Accessed Accessed February 10, 2018

[21] Maharaj P, Cleland J. Integration of sexual and reproductive health services in KwaZulu-Natal, South Africa. Health Policy Plan 2005;20:310–8. doi:10.1093/heapol/czi038.

[22] Winestone LE, Steinfeld RL. Perspectives of providers and clients on Integration of Sexual and Reproductive Health and HIV services in Nyanza Province , Kenya n.d.

[23] Brief TT, Goals MD. Making health systems work 2008:1–10. Accessed February 10, 2018

[24] January F. PEPFAR Guidance on Integrating Prevention of Mother to Child Transmission of HIV , Maternal , Neonatal , and Child Health and Pediatric HIV Services 2011. Accessed January 29, 2018

[25] IATT. Compendium of Case Studies HIV and Sexual and Reproductive Health Programming : Innovative Approaches to Integrated Service Delivery 2014. Accessed February 10, 2018

[26] Gribble J, Foreman MIA. BUREAU MATERNAL AND CHILD HEALTH CARE : 2011;1999.

[27] Health KM of. Minimum Package for Reproductive Health (RH) and HIV Integrated Services 2012. Accessed February 5, 2018

[28] Kenya urban reproductive health initiative (Tupange); Report of the 2010 baseline household survey 2011. Accessed January 29, 2018

[29] Statistics KNB of. 2009 Kenya Population and Housing Census. 2010. Accessed February 5, 2018

[30] Keyonzo N, Nyachae P, Kagwe P, Kilonzo M, Mumba F, Owino K, et al. From Project to Program: Tupange's Experience with Scaling Up Family Planning Interventions in Urban Kenya. Reprod Health Matters 2015;23:103–13. doi:10.1016/j.rhm.2015.06.010.

[31] Muthamia M, Owino K, Nyachae P, Kilonzo M, Kamau M, Otai J, et al. The Tupange Project in Kenya: A Multifaceted Approach to Increasing Use of Long-Acting Reversible Contraceptives. Glob Heal Sci Pract 2016;4:S44–59. doi:10.9745/GHSP-D-15-00306.

[32] USAID, UNICEF W. Towards Universal Access: Scaling up HIV services for women and children in the health sector. Progress Report 2008. 2008. Accessed February 10, 2018

[33] Dieleman M, Harnmeijer JW. Improving health worker performance : in search of promising practices. Hum Resour Health 2006:77.

[34] Mockiene V, Suominen T, Välimäki M, Razbadauskas A. Impact of intervention programs on nurses' knowledge, attitudes, and willingness to take care of patients with human immunodeficiency virus/acquired immunodeficiency syndrome: a descriptive review. Medicina (Kaunas) 2010;46:159–68.

[35] Okundi, B.Aloo-Obunga, C.Sanders, R.Shepherd, C.Green C. Rapid assessment on policy and operational barriers to the integration of FP/RH/HIV services in Kenya | UNESCO HIV and Health Education Clearinghouse. 2009.

Tableau 1 : Description de la population de l'étude – Prestataires de soins

Caractéristique	Médiane (EI)	Nombre	Pourcentage
Âge (ans) : N=103	33 (27 - 44)		
Temps écoulé depuis l'obtention du diplôme (années) : N=103	7 (5-16)		
Expérience professionnelle (mois) : N=103	32 (17 - 38)		
Sexe (féminin)		95	92,2 %
**Cadre**			
Praticien/Médecin généraliste		2	1,9 %
Responsable clinique		4	3,9 %
Personnel infirmier		97	94,2 %
Total		103	100 %

Tableau 2 : Connaissances et compétences des prestataires de soins en matière de planification familiale selon le niveau d'intégration

Description des variables	N	Catégorie 0 : Aucune intégration	Intégration de catégorie 1	Intégration de catégorie 2	valeur p
**A : Prestataires de soins suffisamment informés sur la PF**					
DVC	18	n=8 (0 %)	n=4 (25 %)	n=6 (33 %)	0,275[a]
**Services de prise en charge du VIH/SIDA**	19	n=1 (0 %)	n=4 (0 %)	n=14 (0 %)	Non effectué
SPN	19	n=4 (50 %)	n=13 (69 %)	n=2 (100 %)	0,599[a]
SPoN	20	n= 3 (33 %)	n=1 (100 %)	n=16 (75 %)	0,455[a]
CPE	19	n=8 (75 %)	n=8 (75 %)	n=3 (100 %)	1,000[a]
**B : Formation et compétences du prestataire de soins**					
Compétences pour la fourniture de méthodes de PF à courte durée d'action	103	n=26 (96 %)	n=34 (97 %)	n=43 (93 %)	0,850[b]
Compétences pour la fourniture de méthodes LARC de PF	103	n=26 (85 %)	n=34 (85 %)	n=43 (81 %)	0,944[b]

Description des variables	N	Catégorie 0 : Aucune intégration	Intégration de catégorie 1	Intégration de catégorie 2	valeur p
A reçu une formation à la PF	103	n=26 (92 %)	n=34 (88 %)	n=43 (100 %)	***0,048[b]***
A reçu une formation à la PF au cours des 12 mois précédent l'étude	103	n=26 (81 %)	n= 34 (68 %)	n=43 (72 %)	0,522[b]
A accès aux directives de prestation de services de PF	89	n= 19 (95)	n=30 (83)	n=40 (85)	0,612[b]
A un bon niveau de connaissance des directives de prestation de services de PF	83	n=17 (71 %)	n=28 (65 %)	n=36 (66 %)	0,901[b]
Aborde avec les patients le besoin de planification familiale	95	N=23 (87 %)	N=31 (100 %)	N=41 (80 %)	0,020
**C : Barrière à la prestation de services de PF**					
Pénurie de fournitures / produits de PF éprouvés	103	n=26 (62 %)	n=34 (44 %)	n=43 (30 %)	***0,0382[b]***
Site inadapté – confidentialité	103	n=26 (12 %)	n=34 (3 %)	n=43 (5 %)	0,431[b]
Charge de travail importante	103	n=26 (19 %)	n=34 (24 %)	n=43 (26 %)	0,915[b]

[a]Valeur p test Chi-carré ; [b]Valeur P test exact de Fisher

Tableau 3 : Perception par les patients du temps passé dans les différents domaines de services

Perception de la durée	DVC (N=15)	SPN (N=20)	SPoN (N=16)	CPE (N=31)	Services de prise en charge du VIH/SIDA dans les CSC (N=14)	Moyenne (N=96)
Raisonnable	46,7 %	45,0 %	56,2 %	54,8 %	50,0 %	50,5 %
Courte	20,0 %	10,0 %	12,5 %	0,0 %	7,1 %	9,9 %
Longue	33,3 %	40,0 %	31,3 %	45,2 %	42,9 %	38,6 %
Non indiquée	0 %	5,0 %	0 %	0 %	0 %	1,0 %
Total	100 %	100 %	100 %	100 %	100 %	100 %
