# Supplementary material for: Strengthening integration of family planning with HIV/AIDS and other services: experience from three Kenyan cities
Source: Reprod Health. 2019 May 29;16(Suppl 1):62. doi: 10.1186/s12978-019-0715-8 (PMC6538540; doi:10.1186/s12978-019-0715-8)
Supplement: Supplementary file 2 — Translation of the abstract of this article into Portuguese. (PDF 105 kb) [file 12978_2019_715_MOESM2_ESM.pdf]

Reproductive Health 2019, 16 (Suppl 1) [translated from the original publication]

Reforçar a integração dos serviços de planeamento familiar para utentes com VIH/SIDA e outros serviços: Experiência de três cidades do Quénia

Raymond Mutisya[1*], Jonesmus Wambua[1], Paul Nyachae[1], Mercy Kamau[1], Shalmali Radha Karnad[1], Mark Kabue[2],

[1]Jhpiego Kenya, Nairobi, Kenya
[2]Jhpiego Baltimore, Baltimore, MD, USA

*Contacto do autor correspondente: Raymond Mutisya, Raymond.Mutisya@jhpiego.org; aymondmutisya@yahoo.com
E-mail dos autores:
Jonesmus Wambua, Jonesmus.Wambua@jhpiego.org
Paul Nyachae, Paul.Nyachae@jhpiego.org
Mercy Kamau, Mercy.Kamau@jhpiego.org
Shalmali Radha Karnad, Radha.Karnad@jhpiego.org
Mark Kabue, Mark.Kabue@jhpiego.org

Resumo

Introdução:

O Quénia registou progressos notáveis ao longo deste último ano na integração de uma gama de serviços de saúde reprodutiva com serviços de VIH/SIDA. Este estudo descreve um subconjunto de resultados provenientes do projeto Kenya Urban Reproductive Health Initiative (*Tupange*) (2010 – 2015), fundado pela Bill & Melinda Gates Foundation (BMGF) e liderado pela Jhpiego, que visa especificamente reforçar a integração do planeamento familiar (PF) com uma gama de serviços de cuidados de saúde primários, incluindo aconselhamento e despistagem do VIH, serviços de cuidado do VIH, bem como cuidados maternais, de recém-nascidos e infantis.

Métodos:

Um estudo transversal realizado entre agosto e outubro de 2013 no Quénia por Raymond Mutisya et al. avaliou o nível de integração do planeamento familiar em seis outras áreas de prestação de serviços (cuidados pré-natais, maternidades, cuidados pós-natais, assistência

infantil, aconselhamento e despistagem do VIH, e serviços de VIH/SIDA em contextos de cuidados clínicos abrangentes). As variáveis de interesse foram o nível de integração, as competências e o conhecimento do fornecedor. Os dados de monitorização de programa de rotina sobre a carga de trabalho foram utilizados para amostragem com dados adicionais recolhidos e analisados a partir de vinte instalações de saúde selecionadas para este estudo, incluindo entrevistas de saída de clientes. Foram realizadas análises descritivas, bem como testes do Qui-quadrado/testes exatos de Fisher, para explorar as relações entre as variáveis de interesse.

Resultados:

A integração de PF ocorreu em todas as cinco áreas de serviços, em graus diversos. O conhecimento do fornecedor de serviços sobre PF nas quatro áreas de prestação de serviços (clínicas de aconselhamento, cuidados pré-natais/pós-natais e assistência infantil) aumentou com níveis de integração progressivos. 47% dos clientes referiu que o tempo despendido em aceder aos serviços de PF nas clínicas de aconselhamento foi razoável. Contudo, não foi indicado nenhum conhecimento de PF por parte dos fornecedores de serviços nas clínicas de cuidados abrangentes para VIH/SIDA em todos os níveis de integração, apesar da verificação de disponibilização de aconselhamento e encaminhamento para os serviços de PF.

Conclusões:

A integração de serviços de PF noutras áreas com serviços de cuidados primários, incluindo uma clínica de aconselhamento, pode ser melhorada através de intervenções específicas na instalação. É recomendado e necessário aplicar uma abordagem global para dar resposta à

capacidade e ao comportamento dos fornecedores de serviços, assegurar a segurança dos produtos de PF, bem como criar um ambiente de apoio para acomodar os serviços integrados. Também é necessário realizar estudos adicionais para identificar formas de melhorar a integração de PF, especificamente nos serviços de cuidados de VIH/SIDA.

Palavras-chave: Planeamento familiar, VIH/SIDA, fornecedores de serviços, níveis de integração, necessidades insatisfeitas.

Sobre este suplemento

Este artigo foi publicado como parte da revista científica *Reproductive Health*, Volume 16, Suplemento 1, 2019: Integração Eficaz dos Serviços de Saúde Sexual e Reprodutiva e de Prevenção, Cuidados e Tratamento do VIH na África Subsariana: Onde estão as provas da implementação do programa?
O suplemento foi publicado como uma colaboração entre as revistas científicas *Reproductive Health* e *BMC Public Health.* O conteúdo integral, incluindo as versões em francês, português e inglês, estão disponíveis online:
https://bmcpublichealth.biomedcentral.com/articles/supplements/volume-19-supplement-1
e
https://reproductive-health-journal.biomedcentral.com/articles/supplements/volume-16-supplement-1
